# Identification of the dog orthologue of human MAS-related G protein coupled receptor X2 (MRGPRX2) essential for drug-induced pseudo-allergic reactions

**DOI:** 10.1038/s41598-020-72819-5

**Published:** 2020-09-30

**Authors:** Eri Hamamura-Yasuno, Takuma Iguchi, Kazuyoshi Kumagai, Yoshimi Tsuchiya, Kazuhiko Mori

**Affiliations:** Medicinal Safety Research Laboratories, Daiichi Sankyo Co., Ltd., 1-16-13 Kitakasai, Edogawa-ku, Tokyo, 134-8630 Japan

**Keywords:** Acute inflammation, Drug development, G protein-coupled receptors

## Abstract

MAS-related G protein coupled receptor-X2 (MRGPRX2), expressed in human mast cells, is associated with drug-induced pseudo-allergic reactions. Dogs are highly susceptible to drug-induced anaphylactoid reactions caused by various drugs; however, the distribution and physiological function of canine MRGPR family genes, including MRGPRX2, remain largely unknown. In the present study, we clarified the distribution of dog MRGPR family genes by real-time quantitative PCR and in situ hybridisation. We also investigated the stimulatory effects of various histamine-releasing agents, including fluoroquinolones, on HEK293 cells transiently transfected with dog MRGPR family genes to identify their physiological function. Dog *MRGPRX2* and *MRGPRG* were distributed in a limited number of tissues, including the skin (from the eyelid, abdomen, and cheek), whereas *MRGPRD* and *MRGPRF* were extensively expressed in almost all tissues examined. Histochemical and in situ hybridisation analyses revealed that *MRGPRX2* was expressed in dog connective tissue-type mast cells in the skin. Intracellular Ca^2+^ mobilisation assay revealed that HEK293 cells, expressing dog MRGPRX2 or human MRGPRX2, but not dog MRGPRD, MRGPRF, and MRGPRG, responded to histamine-releasing agents. Our results suggest that dog MRGPRX2 is the functional orthologue of human MRGPRX2 and plays an essential role in drug-induced anaphylactoid reactions in dogs.

## Introduction

Pseudo-allergic drug reactions, including injection-site erythema and swelling, are one of the most commonly observed adverse events associated with intravenous administration of drugs, such as fluoroquinolones, antibacterial agents, and peptidergic drugs^1,2^. In certain cases, these drugs have been reported to induce more serious outcomes such as hypotension and shock-like syndrome^3^. Recently, pseudo-allergic adverse reactions have been demonstrated to be induced through the activation of MAS-related G protein coupled receptor-X2 (MRGPRX2), which is a Gi- or Gq-coupled receptor expressed in human mast cells^4^. The MRGPR family in rodents and humans comprises ~ 40 members and can be divided into several subfamilies (MRGPRA to -H and -X) because of sequence similarities^5–9^. Among rodents and primates, subfamilies A, B, C, and H exist only in rodents, whereas subfamily X is detected in primates, including humans, macaques, and rhesus monkeys^10^. Mrgprb2 and Mrgprb3 are the mouse and rat orthologues of human MRGPRX2, respectively^11,12^. Moreover, functional heterogeneity exists between human MRGPRX2 and mouse Mrgprb2^4,11^. Sabramanian et al*.* have suggested that the Mrgprb2 mutant mouse may not be a suitable model to screen drugs with pseudo-allergic potential for human use^4^. This is because the half-maximum effective concentration (EC_50_) value of Ca^2+^ mobilisation, induced by substance P and fluoroquinolones, in cells transfected with human MRGPRX2 is markedly lower than that of cells transfected with mouse Mrgprb2^4,11^.

Dogs are one of the most commonly used non-rodent species for the evaluation of preclinical toxicity during drug development^13^. In addition, dogs are highly susceptible to drug-induced anaphylactoid reactions including severe hypotension and shock-like syndrome caused by various drugs^14–17^. In fact, several fluoroquinolones, opioids, and neuromuscular blocking agents have been shown to produce severe hypotension in parallel to elevation of blood histamine when administered intravenously in bolus doses to dogs^18–21^. Furthermore, the dose levels of these drugs to induce histamine release and cardiovascular adverse effects were 30- to 100-fold lower in the dog than the rat^16^, suggesting that the dog may be a suitable model for detecting the pseudo-allergic potential including cardiovascular adverse reactions of candidate drugs in the preclinical phase. In dogs, *MRGPRA*, *C*, *D*, *E*, *F*, *G*, and *H*, in addition to *X*2, have been identified so far^22,23^, and among these genes, a total of four genes encoding MRGPR proteins (D, F, G, and X2) have been listed in the National Center for Biotechnology Information (NCBI). More recently, Grimes et al*.* have shown that U2OS cells expressing beagle dog MRGPRX2 responded to compound 48/80 and various peptidergic drugs^24^. However, the localisation and physiological function of dog MRGPR family genes, including MRGPRX2, remain largely unknown.

The present study was designed to identify the functional orthologue of human MRGPRX2 in canine mast cells. We evaluated the distribution of dog MRGPR family genes (*D*, *F*, *G*, and *X*2) in 21 tissues or organs obtained from male beagle dogs by quantitative reverse transcriptional PCR (RT-qPCR). We also investigated the expression of *MRGPRX2* in dog mast cells from several tissues by histochemical and in situ hybridisation (ISH) analyses. Furthermore, we confirmed the stimulatory effects of compound 48/80 and several fluoroquinolones (ciprofloxacin [CPFX], gatifloxacin [GFLX], levofloxacin [LVFX], and pazufloxacin [PZFX]) on HEK293 cells transiently transfected with dog MRGPR family genes or human *MRGPRX2* to identify the physiological function of dog MRGPR family genes by intracellular Ca^2+^ mobilisation assay.

## Results

### Characteristic of MRGPR family in dogs

**Figure 1:**
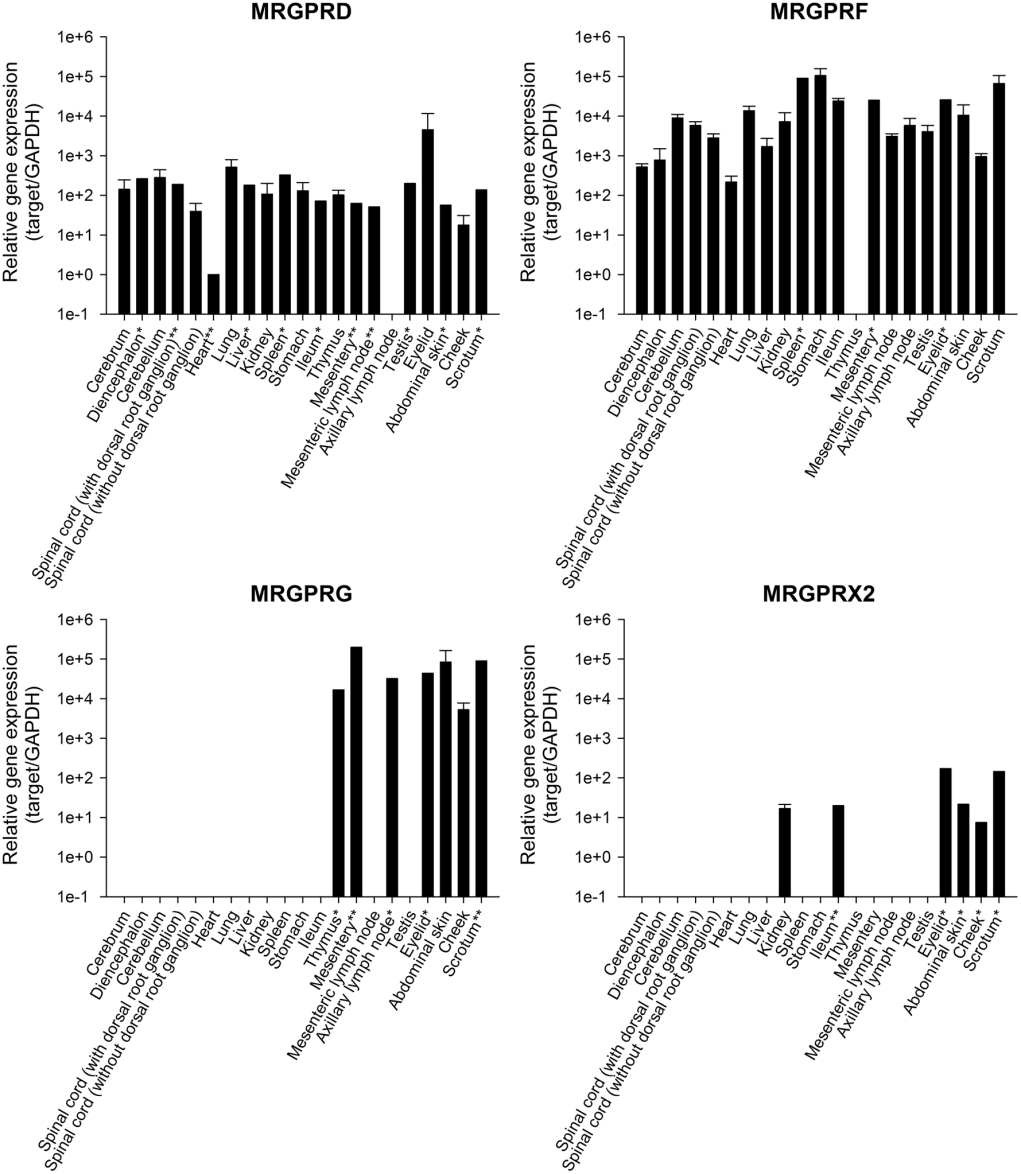
Localisation of dog MRGPR family genes. RT-qPCR analysis was performed to determine MRGPR family gene expression in various canine tissues. Relative gene expression level was calculated by 2^−ΔΔCt^ method, using GAPDH as internal control. The ΔΔCt was calculated by subtracting the ΔCt of MRGPRD in the heart from the ΔCt of each sample. Data are expressed as the mean ± SD of three animals, except for the mean of two animals (*) or individual data of one animal (**) because of Ct > 40 or non-specific amplification.

Homology analysis using BLASTP revealed that dog MRGPRX2 had the highest amino acid sequence homology to human MRGPRX2 (62% sequence homology), whereas dog MRGPRD, F, and G only shared 30–40% sequence identity with human MRGPRX2 (Table 1). Among four dog MRGPR family genes, *MRGPRD* and *MRGPRF* were widely expressed in almost all the tissues (Fig. 1). On the contrary, *MRGPRG* and *MRGPRX2* were not found systemically and were mainly localised to the cutaneous tissues including the eyelid, abdominal skin, cheek, and scrotum (Fig. 1). *MRGPRX2* was also found to be expressed in the kidney and ileum, and *MRGPRG* was found in the thymus, mesentery, and axillary lymph nodes (Fig. 1).

**Table 1:** Amino acid sequence homology between members of dog MRGPR family and human MRGPRX2.

	% Identity	Size (amino acid)	Accession no.
Dog MRGPRD	41	349	XP_540806
Dog MRGPRF	38	343	NP_001300758
Dog MRGPRG	32	286	NP_001300759
Dog MRGPRX2	62	437	XP_005633869
Mouse Mrgprb2	53	338	NP_780740
Rat Mrgprb3	56	247	AAQ08313
Human MRGPRX2	–	330	NP_001290544

Homology analysis (Protein BLAST, BLASTP) was performed using the Basic Local Alignment Search Tool (BLAST, https://blast.ncbi.nlm.nih.gov/Blast.cgi) of the National Center for Biotechnology Information.

### Expression of *MRGPRX2* in mast cells of dogs

**Figure 2:**
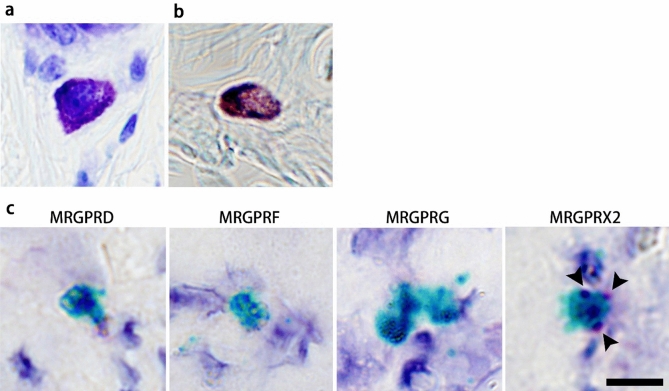
Characteristics of dog skin (cheek) mast cells. Skin mast cells were positive for alcian blue and safranin O and expressed *MRGPRX2*. (**a**) Toluidine blue staining. (**b**) Alcian blue (blue) and safranin O (red) staining. (**c**) In situ hybridisation for *c-kit* (green) and MRGPR family genes (red). No expression of *MRGPRD*, *MRGPRF*, or *MRGPRG* was found in canine skin mast cells. Scale bar: 10 μm.

Histochemical analysis revealed that dog skin mast cells, which showed metachromatic staining with toluidine blue, were positive for both alcian blue and safranin O staining (Fig. 2a,b). Furthermore, ISH showed that dog skin mast cells co-expressed *c-kit* and *MRGPRX2*, but not *MRGPRD*, *MRGPRF*, and *MRGPRG* (Fig. 2c).

### Dog MRGPRX2 is the functional orthologue of human MRGPRX2

**Figure 3:**
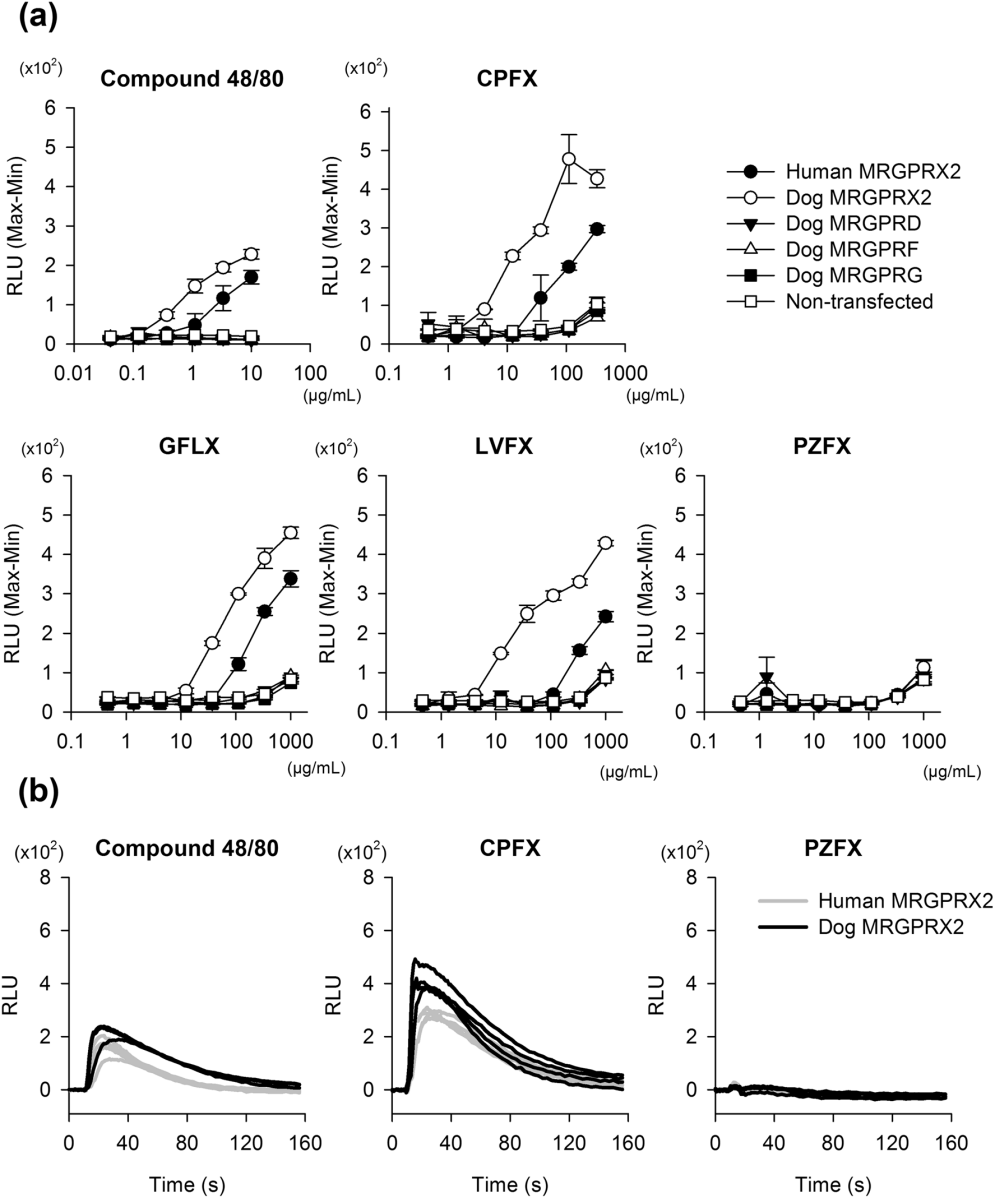
Various histamine-releasing agents activate dog MRGPRX2 and human MRGPRX2 expressed in HEK293 cells. HEK293 cells transiently transfected with dog MRGPR family genes (D, F, G, or X2) or human *MRGPRX2* were exposed to compound 48/80 or fluoroquinolones (ciprofloxacin [CPFX], gatifloxacin [GFLX], levofloxacin [LVFX], and pazufloxacin [PZFX]). (**a**) Increase in intracellular Ca^2+^ levels in a concentration dependent manner. Data represent the mean of quadruplicate assays. Non-transfected HEK293 cells were used as a negative control. (**b**) Time-course changes of intracellular Ca^2+^ levels in dog or human MRGPRX2-expressing HEK293 cells. Traces show representative intracellular Ca^2+^ fluctuation following exposure to compound 48/80 (10 μg/mL), CPFX (333 μg/mL), and PZFX (333 μg/mL). Test articles were perfused from 10 s. *RLU* Relative light units, *s* second.

HEK293 cells transfected with dog *MRGPRX2* responded to compound 48/80, CPFX, GFLX, and LVFX, whereas cells expressing dog MRGPRD, MRGPRF, and MRGPRG did not react to any of the test articles (Fig. 3a). On the contrary, PZFX, which does not induce histamine release in dogs^25^, did not activate dog or human MRGPRX2. The increase in intracellular Ca^2+^ levels, both in HEK293 cells transfected with dog *MRGPRX2* or human *MRGPRX2*, caused by compound 48/80, CPFX, GFLX, and LVFX were concentration-dependent. Interestingly, the EC_50_ values of compound 48/80, CPFX, GFLX, and LVFX to activate MRGPRX2 were lower in dog MRGPRX2-expressing cells compared to those in human MRGPRX2-expressing cells (Table 2). Intracellular Ca^2+^ mobilisation in HEK293 cells expressing dog MRGPRX2 or human MRGPRX2 occurred immediately after treatment with the test articles (Fig. 3b).

**Table 2:** Half-maximum effective concentration (EC_50_) values of test articles on changes in intracellular Ca^2+^ levels in human or dog MRGPRX2-expressing cells.

Test article	EC_50_ (μg/mL) ± SD	
	Human MRGPRX2	Dog MRGPRX2
Compound 48/80	3.0 ± 0.5	1.1 ± 0.4
CPFX	93.5 ± 10.3	14.5 ± 1.8
GFLX	198.5 ± 19.7	78.7 ± 10.9
LVFX	384.7 ± 101.3	58.9 ± 19.9

Data represent the mean ± SD of three independent experiments.

*CPFX* ciprofloxacin, *GFLX* gatifloxacin, *LVFX* levofloxacin.

## Discussion

MRGPR family genes are expressed predominantly in the sensory neurons of the dorsal root ganglia (DRG) and trigeminal ganglia, and mast cells of mammals including rodents and primates^10^. Among MRGPR subfamilies (MRGPRA to H, and X), subfamily A to C and H are present in rodents^10^. MRGPRD to G subfamilies have been reported to be conserved between primates and rodents, and encode one protein per species^5–7^. MRGPRX subfamily exists in primates, and four proteins (MRGPRX1 to X4) are listed in NC-IUPHAR^10^. With regard to dog, which is highly susceptible to drug-induced anaphylactoid reactions, *MRGPRA*, *C*, *D*, *E*, *F*, *G*, and *H*, in addition to *X2*, have been identified so far^22,23^. In the present study, we characterised four dog MRGPR subfamilies (MRGPRD, MRGPRF, MRGPRG, and MRGPRX2), which were listed in the NCBI database. *MRGPRD* and *MRGPRF* were expressed in a variety of tissues and organs in dogs. In rodents, *Mrgprd* and *Mrgprf* have been reported to be expressed in a relatively limited number of tissues; *Mrgprd* was localised to the DRG, urinary bladder, testis, uterus, and arteries^26^, and *Mrgprf* was mainly distributed in the vas deferens, uterus, intestine, stomach, and aorta^27^, suggesting that dog MRGPRD and MRGPRF have distinct distribution characteristics from rodents. However, *Mrgprd* was reported to be expressed in aortic endothelial cells and leukocytes, including neutrophils, macrophages, and lymphocytes, in rodents^28–30^. Furthermore, human *MRGPRF* was identified in enteroendocrine cells^31^, in addition to enteric neurons^32^. We did not evaluate the localisation of dog *MRGPRD* and *MRGPRF* at the cellular level in this study, and hypothesise that they might be expressed in various cells or tissues, including arteries, blood cells, or enteroendocrine cells, on the basis of the information gathered. In this study, *MRGPRG* and *MRGPRX2* were confirmed to be expressed in a limited number of tissues, including the skin (eyelid, abdomen, and cheek) of dogs. In the Genotype-Tissue Expression (GTEx) project, human *MRGPRG* has been reported to show high expression in the testis (GENE Code ID. ENSG00000182170.3, GTEx Analysis Release V8), where expression of dog *MRGPRG* was not detected in our study. Human *MRGPRX2* has been shown to be highly expressed in the skin, adipose tissue, bladder, and colon^12^, and mast cells, sensory neurons, and keratinocytes^5,33,34^. The distribution profile of dog *MRGPRX2* appeared to be consistent with that of human *MRGPRX2*, at least partially. In our study, quantitative PCR analysis showed that the expression levels of dog *MRGPRX2* appeared to be lower than those of *MRGPRD*, *MRGPRF*, and *MRGPRG*; therefore, the expression profile of dog MRGPRX2 should be further investigated.

Dog MRGPRX2 has relatively higher sequence homology (62%) to human MRGPRX2 than that of rodents; homologies of Mrgprb2 and Mrgprb3, the human MRGPRX2 orthologues of mice and rats, are 53% and 56%, respectively. One can hypothesise that lower homologies between rodent and human MRGPR proteins result in different sensitives to drugs. In fact, McNeil et al. demonstrated that the EC_50_ of substance P in mouse Mrgprb2-expressing cells is 360-fold higher compared to that in human MRGPRX2-expressing cells (mouse: 54 μM, human: 152 nM)^11^. Therefore, it is crucial to select appropriate animal species to predict drug-induced pseudo-allergic reactions in humans.

As is the case with rodents^35^, dog mast cells are classified into two types; the mucosal-type mast cell (MMC) or connective tissue-type mast cell (CTMC)^16,17^. Histochemical analysis revealed that mast cells present in the skin from the cheek were positive for both alcian blue and safranin O, indicative of CTMC, in line with our previous work, which responded to basic secretagogues^16,17^. Furthermore, CTMC in the skin expressed *c-kit* and *MRGPRX2*, indicating that canine CTMC in the skin expressed the human MRGPRX2 orthologue.

More recently, Grimes et al*.* reported that compound 48/80 and several types of peptides activated dog MRGPRX2^24^. Consistent with their research^24^, we also confirmed that compound 48/80 activated MRGPRX2, but not MRGPRD, F, and G, suggesting that dog MRGPRX2 expressed in canine CTMC is the functional orthologue of human MRGPRX2. In human MRGPRX2-expressing cells, the EC_50_ of certain drugs, such as compound 48/80, somewhat varied among the facilities^11,12,24^. This might have resulted from the difference in the host cells used, transfection methods, and co-expressed Gα proteins. For example, EC_50_ of compound 48/80 to activate human MRGPRX2 ranges from 0.47–3.75 μg/mL^11,12,24^. Grimes et al*.* reported that the EC_50_ of compound 48/80 for activating human MRGPRX2 with Gα_16_ and dog MRGPRX2 without Gα_16_ in their assay platform, was comparable^24^. However, the EC_50_ value of compound 48/80 in dog MRGPRX2-expressing cells was *ca.* three times lower than that in human MRGPRX2-expressing cells in this study. Therefore, further investigations are necessary to clarify the factors affecting distinct reactivity among assay platforms.

Dog MRGPRX2 was also activated with several fluoroquinolones, including CPFX, in the present study. The EC_50_ values of CPFX and LVFX required to activate dog MRGPRX2 were approximately 15 μg/mL and 60 μg/mL, respectively. These values were comparable to results in our previous report, where CPFX and LVFX induced histamine release from dispersed canine skin mast cells at 10 μg/mL or more and 30 μg/mL or more, respectively^16^. In addition, the EC_50_ value of CPFX required to activate human MRGPRX2 was approximately 100 μg/mL, which was comparable to the concentration (200 μg/mL) required to induce histamine release from dispersed mast cells of human skin^36^. Further, PZFX does not cause histamine release even in the dog, the most susceptible species to fluoroquinolones^25^. In the present study, PZFX did not activate dog MRGPRX2, indicating our assay platform using HEK293 cells expressing dog MRGPRX2 could mimic the histamine release assay using dispersed dog mast cells.

The difference in EC_50_ values of compound 48/80 between dog MRGPRX2 and human MRGPRX2 was smaller (*ca.* threefold) than the values of CPFX or LVFX (*ca.* sevenfold) in this study, suggesting that there might be differences in ligand selectivity between dog and human MRGPRX2. A basic substituent at position 7 of fluoroquinolones is suggested to associate with its histamine-releasing property^37^. Thus, it might be valuable to compare the interaction between the basic substituent at position 7 of each fluoroquinolone and each receptor by structure–activity relationships to elucidate the factors causing differences in EC_50_ values between human and dog proteins. Recently, it has been reported that the binding of compound 48/80 or substance P to MRGPR genes was remarkably lowered by replacing the amino acid at position 164 of human MRGPRX2 and the amino acid at position 171 of murine Mrgprb2, which are located in equivalent tertiary structural regions^38^. To clarify the factors contributing to species-based differences in sensitivity, further investigation on the structures of the receptors, including comparisons of amino acid sequences and tertiary structures, and the identification of binding sites using recombinant mutants is required.

It has been reported that human and dog mast cells showed high similarity; protease content and histamine releasing property including degranulation process by several drugs to date^39^. In addition, as we have shown in this study, dog MRGPRX2 has high similarities to human MRGPRX2 both in distributions and functions. Therefore, in vivo and in vitro systems using dogs have the potential to be the best models for elucidating and predicting the mechanisms of mast cell-related adverse effects in human clinical.

In summary, dog *MRGPRX2* was distributed in a limited number of tissues, including the skin, similar to human *MRGPRX2*, and expressed in CTMC. Basic secretagogue compound 48/80 and fluoroquinolones activated dog MRGPRX2, indicating that dog MRGPRX2 is the functional orthologue of human MRGPRX2. As shown here, dog MRGPRX2 was found to be highly susceptible to certain drugs, including fluoroquinolones. Based on the similarity between human and dog mast cells including MRGPRX2, and on the susceptibility of dogs to anaphylactoid reactions, dog is a suitable model to predict the potential risk for human use and elucidate the mechanism of drug-induced pseudo-allergic reactions.

## Materials and methods

### Homology analysis of dog MRGPR family with human MRGPRX2

Homology analysis of the amino acid sequence (Protein BLAST, BLASTP) was carried out using Basic Local Alignment Search Tool (BLAST, https://blast.ncbi.nlm.nih.gov/Blast.cgi) of NCBI.

### Reagents

Compound 48/80 was purchased from Sigma-Aldrich Co. LLC. (St. Louis, MO, USA). CPFX and LVFX were obtained from Fujifilm Wako Pure Chemical Corporation (Osaka, Japan), and GFLX and PZFX were obtained from LKT Laboratories Inc. (St. Paul, MN, USA).

### Animals

A total of three male beagle dogs were purchased from Marshall BioResources Japan (Tsukuba, Japan). The animals weighing 10–13 kg were 2–3 years old. The animals were housed individually in stainless steel cages with a controlled temperature of 18–28 °C and humidity of 30–70%, and a 12-h light (from 07:00 to 19:00, 300 luces or more) and 12-h dark cycle. Certified canine diet (CD-5M, Clea Japan, Inc., Tokyo, Japan) and chlorinated water were provided to each animal ad libitum. The experimental protocol was approved in advance by the Ethics Review Committee for Animal Experimentation of Daiichi Sankyo Co., Ltd. (Tokyo, Japan). All animal procedures were performed in accordance with the guidelines of the Animal Care and Use Committee of Daiichi Sankyo Co., Ltd.

### Sampling of the tissues/organs

Animals were euthanised humanely under anaesthesia with an intravenous injection of sodium pentobarbital (25 mg/kg, Somnopentyl Injection, Kyoritsu Seiyaku Corporation, Tokyo, Japan). The brain (cerebrum, diencephalon, and cerebellum), spinal cord (lumbar, with or without dorsal root ganglion), heart (ventricular papillary muscle), lung (right lower lobe), liver (left lateral lobe), kidney, spleen, stomach, ileum, thymus, mesentery, mesenteric lymph node, axillary lymph node, testis, eyelid, abdominal skin, cheek, and scrotum were collected. A portion of each tissue (approximately 200 mg) was excised, snap-frozen in liquid nitrogen, and stored at − 80 °C until RNA extraction. The remaining tissue samples were fixed in 10 vol% neutral buffered formalin and embedded in paraffin for immunohistochemistry and in situ hybridisation.

### Quantitative reverse transcription PCR (RT-qPCR)

Total RNA was extracted from each frozen tissue sample using RNeasy Mini Kit (QIAGEN, Hilden, Germany) or TRIzol Reagent (Thermo Fisher Scientific Inc.) with RNase-Free DNase Set (Thermo Fisher Scientific Inc.), according to the manufacturer's protocol. The cDNA samples were synthesised using High-Capacity cDNA Reverse Transcription Kit (Thermo Fisher Scientific Inc.). Expression of dog *MRGPRD*, *MRGPRF*, *MRGPRG*, *MRGPRX2*, and *GAPDH* was analysed using the 7900HT Fast Real Time PCR System (Thermo Fisher Scientific Inc.) with the Fast SYBR Green Master Mix (Thermo Fisher Scientific Inc.) and primer pairs for each gene (see Table 3). qPCR amplifications were performed in duplicate as follows: initial denaturation at 95 °C for 20 s, followed by 40 cycles of amplification at 95 °C for 1 s and 60 °C for 20 s. The results of the target genes (*MRGPRD*, *MRGPRF*, *MRGPRG*, and *MRGPRX2*) were normalised against *GAPDH*. Gene expression levels were represented as relative gene expression using 2^−ΔΔCt^ method. The ΔΔCt was calculated by subtracting the ΔCt of MRGPRD in the heart from the ΔCt of each sample.

**Table 3:** Sequences of the primer pairs for dog MRGPR family genes for RT-qPCR.

Target gene (Accession no.)	Forward primer 5′–3′	Reverse primer 5′–3′
Dog *MRGPRD *(XM_540806)	GGAAGTCCTACATGGCATTG	CCAGATCACCAGGCTGTTC
Dog *MRGPRF *(NM_001313829)	GAGATGGTGGGGAACTGTTC	GAGACAAAGGAGCAGGAAGATG
Dog *MRGPRG *(NM_001313830)	CCTTCACCAACGTGCTCTTC	GAAGCCGAGGAACAGGAAG
Dog *MRGPRX2 *(XM_005633812)	GACGCTGCAGTCACAGTC	CTGGTCACTTGCATTCTTTG
Dog *GAPDH *(NM_001003142)	GGTCGGAGTGAACGGATTTG	GGAACATGTACACCATGTAGTTGAG

### Histochemical analysis and in situ hybridisation

Histochemical analysis and in situ hybridisation were conducted using skin samples, based on the present distribution study of *MRGPRX2* and our previous study using dispersed mast cells^17^. The tissue specimens were stained with 0.1% toluidine blue (pH 3) or 0.1% alcian blue (pH 0.3) and 0.1% safranin O (pH 1). ISH was performed using the RNAscope^®^ 2.5 HD Duplex Reagent Kit and RNAscope probes obtained from Advanced Cell Diagnostics Inc. (Newark, CA, USA), according to the manufacturer’s instructions. Signals for *c-kit* were detected with horseradish peroxidase-based green chromogens, and signals for dog MRGPR family (*D*, *F*, *G*, and *X2*) were detected with alkaline phosphatase-based Fast Red chromogens.

### Transfection of HEK293 cells with dog or human MRGPR genes

HEK293 cells obtained from the JCRB Cell Bank (Osaka, Japan) were transfected transiently with dog MRGPRD, MRGPRF, MRGPRG, or MRGPRX2 or human MRGPRX2. Lipofectamine 2000 Reagent (Thermo Fisher Scientific Inc.) and pcDNA3.1(+) vector containing each gene were diluted and mixed using Opti-MEM I Reduced Serum Medium (Thermo Fisher Scientific Inc.) to prepare lipid-DNA complexes (final concentrations: lipofectamine 2.5 μL/mL and DNA 2,500 ng/mL). HEK293 cells were detached using TrypLE™ Express (Thermo Fisher Scientific Inc.) and prepared to 7 × 10^5^ cells/mL with the lipid-DNA complex. Thereafter, 25 μL cells (1.75 × 10^4^ cells/well) were seeded per well in 384-well flat-bottomed plates (Corning Incorporated, Corning, NY, USA) and incubated overnight at 37 °C under 5% CO_2_ conditions. Cells treated with plasmid-free lipid solution were used as negative control (non-transfected cells).

### Ca^2+^ mobilisation assay

Test articles were dissolved in HBSS (pH 7.4, Thermo Fisher Scientific Inc., Waltham, MA, USA) supplemented with 20 mM HEPES (Sigma-Aldrich Co. LLC.) and 0.05 vol% bovine serum albumin (BSA, Sigma-Aldrich Co. LLC.). The highest concentration of the fluoroquinolones was set at 1000 μg/mL based on previous reports at which the test substances induced marked intracellular Ca^2+^ mobilisation in human MRGPRX2-expressing HEK293 cells^11^ or caused histamine release in rat or human mast cells^16,25,36^. Intracellular Ca^2+^ levels were analysed using Calcium Kit II-iCellux (Dojindo Molecular Technologies, Inc., Kumamoto, Japan), according to the manufacturer's instructions. HEK293 cells (1.75 × 10^4^ cells/well) were loaded with 1.25 mM probenecid and calcium probe for 45 min at 25 °C. Changes in fluorescence intensities before and after addition of the test articles were measured over time using FLIPR Tetra (Molecular Devices, LLC., Sunnyvale, CA, USA) with excitation at 470–495 nm and emission at 515–575 nm. The test articles were added at 10 s after beginning the measurements. Samples were measured in duplicate or quadruplicate. The data were analysed using ScreenWorks (Molecular Devices, LLC. Version 3.2.0.14) to determine the difference between maximal and minimal fluorescence intensity (max–min). As CPFX (1,000 μg/mL) induced nonspecific increase in intracellular Ca^2+^ levels in non-transfected cells, these data were excluded from the analysis.

### Statistical analysis

Data represent the mean ± SD of three animals for distribution study or three independent assays for Ca^2+^ mobilisation. EC_50_ of each test article used in the Ca^2+^ mobilisation assay was calculated using the sigmoid Emax model. These analyses were performed by using the SAS System Release 8.2 software (SAS Institute Inc., Cary, NC, USA).

## Data Availability

All relevant data are present within the paper.
